# Utilizing multi-temporal thermal data to assess environmental land degradation impacts: example from Suez Canal region, Egypt

**DOI:** 10.1007/s11356-022-22237-z

**Published:** 2022-08-05

**Authors:** Mohamed O. Arnous, Basma M. H. Mansour

**Affiliations:** Faculty of Science, Geology Department, Suez Canal University, Ismailia, Egypt

**Keywords:** LULC, LST, Environmental impacts, Waterlogging, Salinization, Seismic activity, Water pollution

## Abstract

Land surface temperature (LST) analysis of satellite data is critical for studying the environmental land degradation impacts. However, challenges arise to correlate the LST and field data due to the constant development in land use and land cover (LULC). This study aims to monitor, analyze, assess, and map the environmental land degradation impacts utilizing image processing and GIS tools of satellite data and fieldwork. Two thermal and optical sets of Landsat TM + 5 and TIRS + 8 data dated 1984 and 2018 were used to map the thermal and LULC changes in the Suez Canal region (SCR). The LULC classification was categorized into water bodies, urban areas, vegetation, baren areas, wetland, clay, and salt. LULC and LST change detection results revealed that vegetation and urban areas increased in their areas in 34 years. Moreover, 97% of the SCR witnessed LST rise during this period with an average rise rate of 0.352 °C per year. The most effective LULC class changes on LST were the conversions from or to baren areas, where baren areas were converted to 630.5 km^2^ vegetation and 104 km^2^ urban areas rising the LST to 43.57 °C and 45 °C, respectively. The spectral reflectance (LSR), LST profiles, and statistical analyses examined the association between LST and LULC deriving factors. In combination with field observations, five hotspots were chosen to detect and monitor natural and human land degradation impacts on LST of the SCR environment. Land degradations detected include water pollution, groundwater rising, salinity increase, sand dune migration, and seismic activity.

## Introduction

Globally, environmental land degradation impacts assessment is crucial for the promising economies and societal development as they drive ongoing sustainable development. Anthropogenic and natural activities are operating not only in the present but also with the past on the surface of the earth as a key driving force of cultural, social, economic development inducing significant environmental changes including water, air pollution, and greenhouse gas emissions in the terrestrial ecosystem at various local, regional, and global scales (Zhang et al. 2017; Choudhury et al. 2019). These changes affect the degree of absorption of solar radiation, albedo, evaporation rates, heat transmission to the soil, heat storage, and wind turbulence and rise the land surface temperature (LST) rapidly (United Nations 2010; Pal and Ziaul 2017; Heinemann et al. 2020). Increasing the demand for the application of modern technology to define, manage, and incorporate environmental land degradation processes is, therefore important for the overall sustainable development of any country’s prerequisites. Also, satellite-based studies will help developers provide recommendations for building design and landscaping major infrastructure projects that are useful in reducing heat accumulation and land use/land cover (LULC) surface retention. There have been significant developments in the appraisal of land surface environmental circumstances from space-borne satellite data, mostly from thermal infrared remote sensing data (Arnous and Green 2015). Remote sensing (RS) and geographic information systems (GIS) technologies have demonstrated their value in mapping patterns in LULC and related environmental impacts with better spatial coverage and in near real time (Lilly Rose and Devadas 2009; Arnous et al. 2011; Sahana et al. 2016; El-Rayes et al. 2017; Arnous et al. 2017). RS and GIS integration have been effective in tracking and analyzing LULC growth patterns and assessing LULC effects on surface temperature. The digital image classification has demonstrated its ability to provide detailed information on the extent, rate, and position of LULC expansion and its related LST rise, especially when coupled with GIS. The alteration of the transition in LULC classes on spectral signature and land surface emissivity can thus be detected (Sahana et al. 2016). Also, the multi temporal RS images can serve as a database for monitoring LULC changes and their dynamics over various time scales in vegetation, water, urban areas, baren lands, salt-affected areas, and wetlands and thus, modeling the dynamics of environmental land surface degradation impacts indicators (Igun and Williams 2018; Choudhury et al. 2019).

Environmental and sustainable development of the Suez Canal region (SCR), Egypt has been highlighted over the past three decades in the narrow Nile Valley and Delta to relieve the population burden. Therefore, Egypt’s government has launched a megaproject called the development project of the century to sustain SCR by turning this investigated area into a major economic zone and prosperous waterfront region, hence increasing the role of the SCR in international trading and developing the Suez Canal Province. In the past decades, many natural and human made environmental hazards namely seismic, groundwater level rising, water pollution, and soil salinization affected the area under investigation and lead to significant environmental land degradation impacts (Ghodeif et al. 2013; Arnous and Hassan 2015; Mansour 2015; Arnous et al. 2015, 2017; Geriesh et al. 2015a, b; El-Rayes et al. 2017; Geriesh et al. 2019). Therefore, SCR’s environmental assessment is important for the management of this important region to address the prevailing hazards, focusing on soil-related issues, climate, tectonic systems, seismicity, and water contamination in response to land degradation. These hazards usually have a great influence on the LST over time. For example, there is a thermal build-up near tectonically active epicentral areas related to earthquakes (Zoran et al. 2016). Therefore, applying RS and GIS tools to identify, monitor, evaluate, assess, and map the environmental hazards and LU/LC environmental response change on LST is significant to enhance sustainable development planning within any area worldwide. In the present study, the monitoring and assessing of these environmental land degradation hazards/impacts will be accomplished with the utility of RS and GIS tools to define the distribution, nature, magnitude, spatial extent, and temporal activities of land degradation as the source to successful prevention and recovery measures. Several RS, GIS-based, and statistical analyses of multi-temporal thermal satellite data and the thematic mapping of urban, agricultural activities, groundwater level rising, salt-affected areas, active seismic fault zones, and water pollution hazards were proposed. Finally, the environmental land degradation prints’ assessment utilizing image processing and GIS tools of multi-temporal space-borne thermal data and fieldwork verification were addressed.

### Study area characteristics

**Fig. 1:**
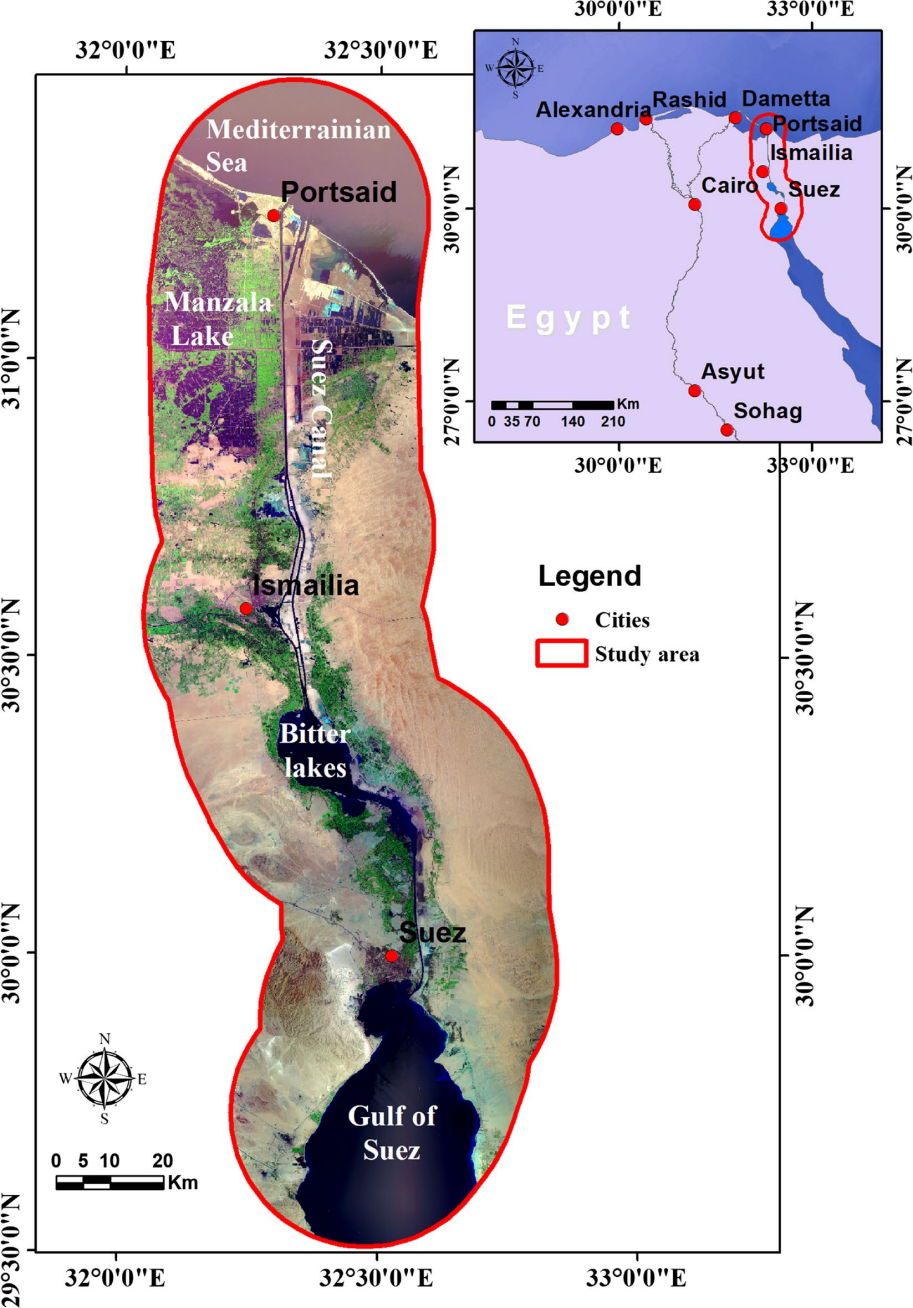
The location map of the Suez Canal region (SCR)

The study area covers about 9920 km^2^. It is located in the northeastern part of Egypt by geo-coordinates 29° 31′′–31° 28′′ N and 32° 03′′–32° 50′′ E. It is bordered by the Mediterranean Sea to the north and by G. Khashm El-Galala and G. Ataqa to the south. The investigated area extends 25 km to the west and the same distance to the east of the Suez Canal corridor (Fig. 1). The location of the study area is characterized by extensive water surfaces mainly the Gulf of Suez rift, Bitter Lakes, Lake El Timsah, and Lake Manzala (LM) as tectonic depressions responsible for most of the World’s Lakes (Holmes 1965). SCR is covered by sedimentary rocks belonging to the Cretaceous up to the Pleistocene age. It is characterized mainly by geomorphological features such as high mountains, slopes, lowlands, lakes, waterlogged areas, marshes sabkhas, drainage, sand dunes, and wadi depression features. The mountainous areas are relatively small and extend from the southern part of the study area to G. Shabrawet northwards. It is steep slopes at the north and east that transform into more gentle–slope terrain to the southwest. The terrain is undulated and dissected by the drainage pattern at G. Ataqa (855 m). The study area’s terrain surface is beginning to elevate with G. Geneifa (265 m) and G. Shabrawet (226 m). An arid climate characterizes the SCR: hot, dry, and rainless in the summer and mild with some showers in winter. It has mean monthly temperatures ranging from 13 °C to 29 °C in the winter and summer season. Meanwhile, the average annual rainfall of this region ranges between 0.3 and 0.5 cm. The daily evaporation varies from approximately 1.5 mm to 7.5 mm in December and July, respectively. The evaporation from the surface open water is about 1600 mm/year (Egyptian Meteorological Authority 2006).

## Materials and methods

**Fig. 2:**
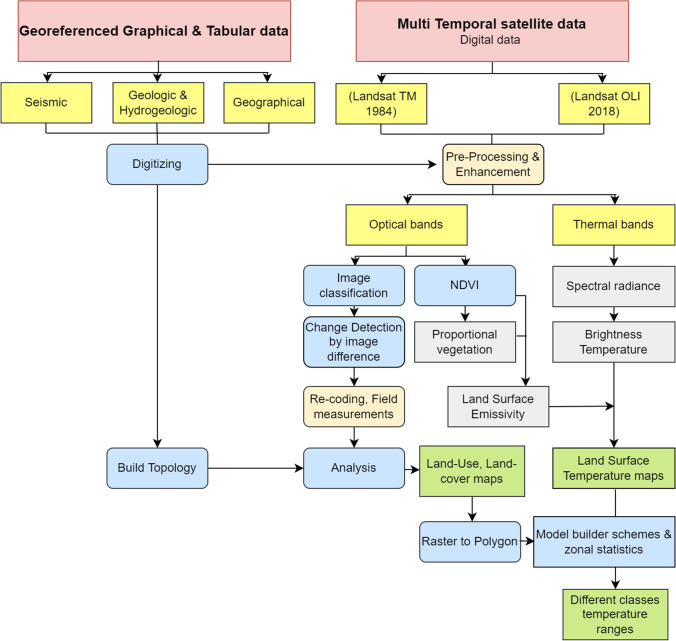
Methods used to reach the study aim

The data used in the current study are RS data (Landsat satellite images) and geo-referenced graphical and tabular data (geological, hydrogeologic, geomorphologic maps, and seismic, field and laboratory measurements). These data are geo-referenced to the UTM coordinate system, zone 36 North, based on topographic map at a scale 1:100,000 generated by the (Egyptian Military Survey 1987). The seismic data over the southern part of the study area during the period 1997–2017 was obtained from the National Earthquake Information Centre (NEIS), the International Seismological Centre (ISC), and the Egyptian National Seismic Network (ENSN). To assess the LULC changes, land degradation and estimate the effects of these changes on LST, the digital image processing methods were used to enhance multi-temporal Landsat series (Fig. 2).

**Fig. 3:**
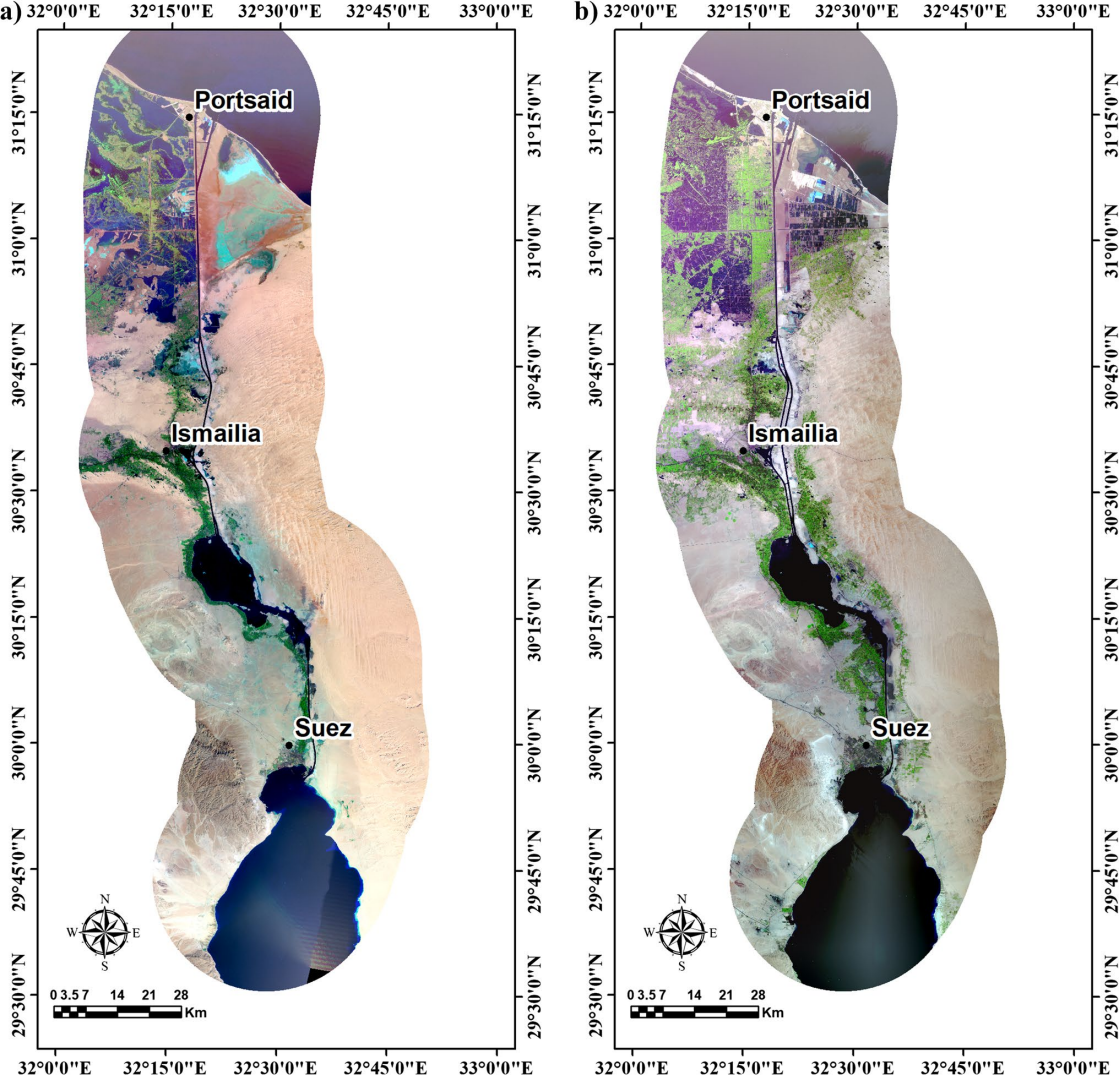
Multi-temporal data is represented by three full scenes of each **a** TM + 4 and **b** OLI + 8

The used multi-temporal data is represented by three full scenes of each TM + 4 and OLI + 8 and TIRS + 8 images, scene numbers (path/row = 175–38, 175–39, and 174–40), dated 1984 and 2018 respectively captured in the summer season in June (Fig. 3a and b). The thermal infrared bands of these data (TM data the wavelength used for LST are band 6, while TIRS + 8 of bands 10 and 11) show the infrared radian’s quantity emitted from dissimilar land surfaces. The images were enhanced utilizing ENVI, ERDAS imagine, and ArcGIS software. The atmospheric correction was done to reduce the noise effect using the FLAASH module. The multi-temporal RS data was radiometrically and geometrically corrected to precise the uneven sensor reaction over the image, and correct the satellite’s geometric distortion data (Arnous and Green 2015). Image enhancement, including contrast stretching, best band combination, and principal component analysis, were used to produce high spectral resolution images required to detect and monitor land degradation environmental impact hazards from 1984 to 2018 (Fig. 2).

Moreover, the multi-temporal RS data was used for image classification of LU/LC into seven classes: water bodies, urban, agricultural land, barren land, wetland, clay, and salt crust by using ENVI and ArcGIS software. In the present study, a supervised image classification of the parametric rule has been applied depending on the pixel with maximum likelihood classification (Pal and Ziaul 2017; Hua and Ping 2018). Moreover, the pixels derived from the multi-temporal classified satellite images were utilized to compare similar sites during the field observation and field measurements. Accuracy assessment of the supervised multi-temporal satellite data was carried out, which provide the overall accuracy of the classified map and each class within it. The change detection techniques were used to carry out a qualitative and quantitative evaluation for the multi-temporal thermal and LULC classified images. In the present study, the post-classified comparison technique is applied for two different classified images within timespan of 34 using model builder GIS tool which gives similar results to cross-tabulation but is much faster in obtaining the temperature ranges for each LULC class. The overall LULC changes are calculated to show the rate of changes for each LULC class and its environmental impacts.

### Estimation and construction of the multi-temporal LST maps

The band pixel has a digital number (DN) value in raw RS data connected to a raw measure the sensor acquires. Therefore, the DN must be converted into physical quantities, radiance, and brightness temperature to obtain quantitative information from raw satellite data.

Correcting the previously calibrated images by atmospheric effects is essential because it can cause significant distortions in the radiometric signal. Hence, the methods used to measure, extract, and map LST from RS satellite data’s thermal bands were followed by detecting the geospatial relationships between SCR changes LULC and LST. The detection of the final LST map was done by the measurement of the normalized difference vegetation index (NDVI), then the conversion of TIRS band data to TOA spectral radiance, and finally, the measurement of atmospheric brightness. LST data were then converted from K to °C (Khyami 2021).

### Statistical analyses

The cluster analysis classification technique was used and is generally placing objects into homogeneous groups to reveal the relation between them. It considers a similarity matrix developed from the new data matrix and defines pair by pair the inter-correlation of variables (*R*-mode) or cases (*Q*-mode) in a two-dimensional hierarchical diagram called the dendrograms Harbuaugh and Marreium (1968). After comparing the LST images and the LULC classification images and determining the temperature ranges of each LULC class using the zonal statistics tool in ArcGIS desktop 10.3 (Fig. 2), the change behavior and relationship between the LULC classes of 1984 and 2018 can be clearly understood using the cluster analyses. The cluster analysis done is based on the Ward method, where the distance metric is the Euclidean distance and depends on the maximum, minimum, and mean values of temperatures in each given LULC class.

The determination of the actual spatial changes within each LULC class and their effect on LST involves specific spatial data processing, where the geoprocessing workflows must be documented. GIS technology provides geoprocessing tools to carry out these specific spatial analyses. GIS spatial models constructed by model builder tool in ArcGIS desktop facilitate the process by automating the workflows. It connects the tasks and functions, allowing the workflow’s execution, modification, and repetition with a single move, increasing the geoprocessing efficiency (Goodchild 2005).

**Fig. 4:**
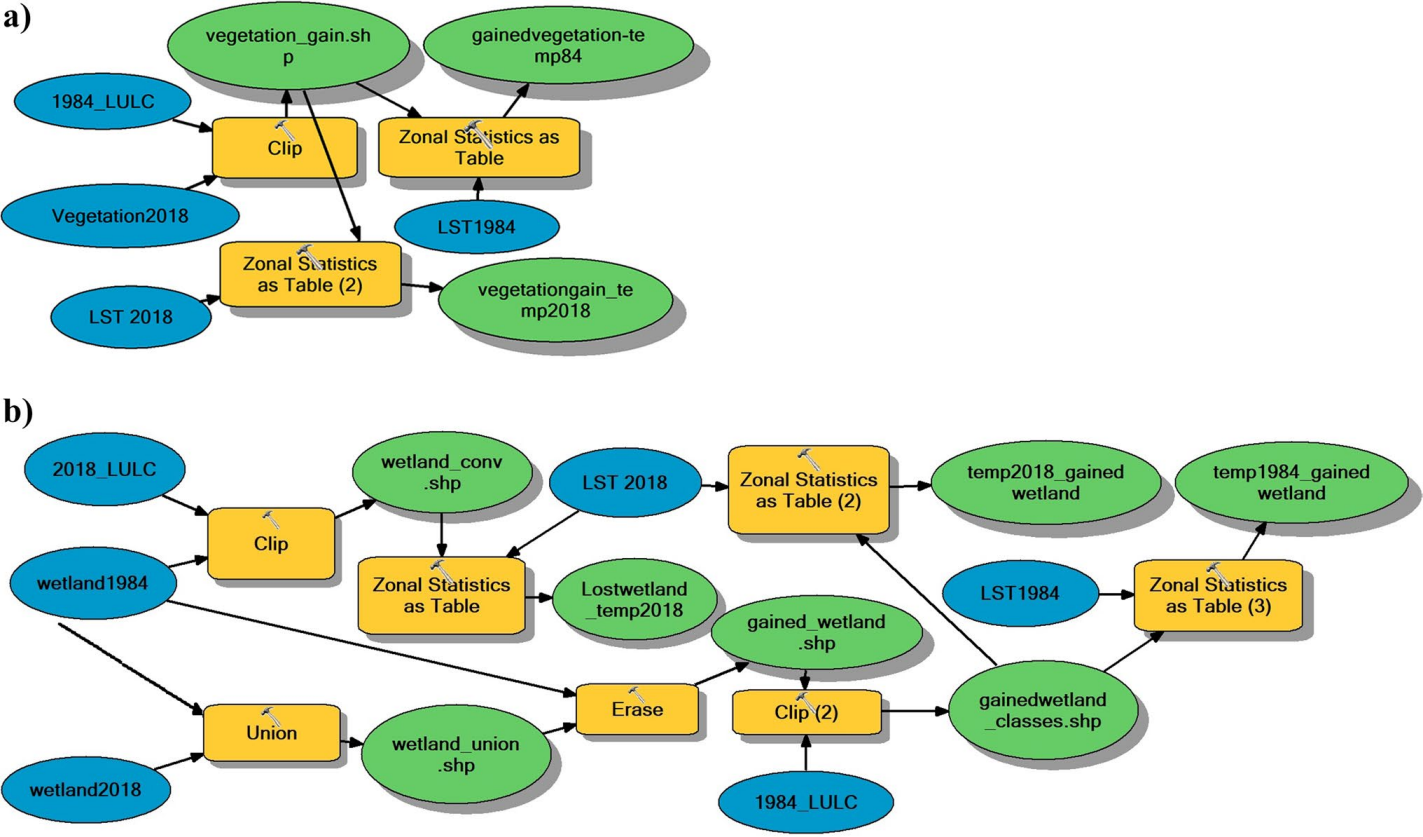
Examples of model builder scheme for obtaining the areal statistical analysis for **a** + changed classes and **b** − changed classes and their effect on LST

To determine these spatial changes within each LULC class from 1984 to 2018 and their effect on LST, each class was analyzed statistically differently according to whether its areal change was positive or negative using the Model builder in ArcGIS 10.3 (Fig. 4). For the classes which increased in area in 2018 (+ change) (Fig. 4a) and those which decreased in area in 2018 (− change) (Fig. 4b), the analysis was done in the ArcGIS by clipping the class polygon in 2018 with the LULC map of 1984 and the class polygon in 1984 with 2018 LULC map, respectively. Thus, we can know the areal contribution of the other classes to this class. This areal contribution was then compared using zonal statistics tool to the LST maps of 1984 and 2018 to show how the conversion of the other classes to the class under investigation changed the LST values from 1984 to 2018 and which of the classes had the most influence.

It was observed that some pixels containing the investigated class with (− change) were added in 2018 and were not present in 1984. So, in this case, there was a class gain. To analyze the class gain in pixels, the class polygon of 1984 and that of 2018 were unioned and then, the class polygon of 1984 was subtracted from the union. The difference was then compared using zonal statistics to the LULC map of 1984 and then to the LST maps of 1984 and 2018.

Across the SCR area, four profiles have been made to represent the variations of LST and LULC reflectance between all major classes between 1984 and 2018. Moreover, according to the type of environmental hazards and land degradation, the SCR was divided into five hot spots, showing the change in the extent and spatial distribution within 34 years of the satellite data due to the natural (seismic, sand dune migration) and/or human activities, particularly agricultural, industrial, and urban activities. Field measurements also included water level and salinity surveys for 5 points east of Bitter lakes to confirm the results deduced from LULC and LST changes by comparing them with previous literature (Geriesh 2004). Both electric sounder and EC meter were used to make water level and salinity surveys, respectively.

## Results and discussion

### LULC types and areal change analysis

**Fig. 5:**
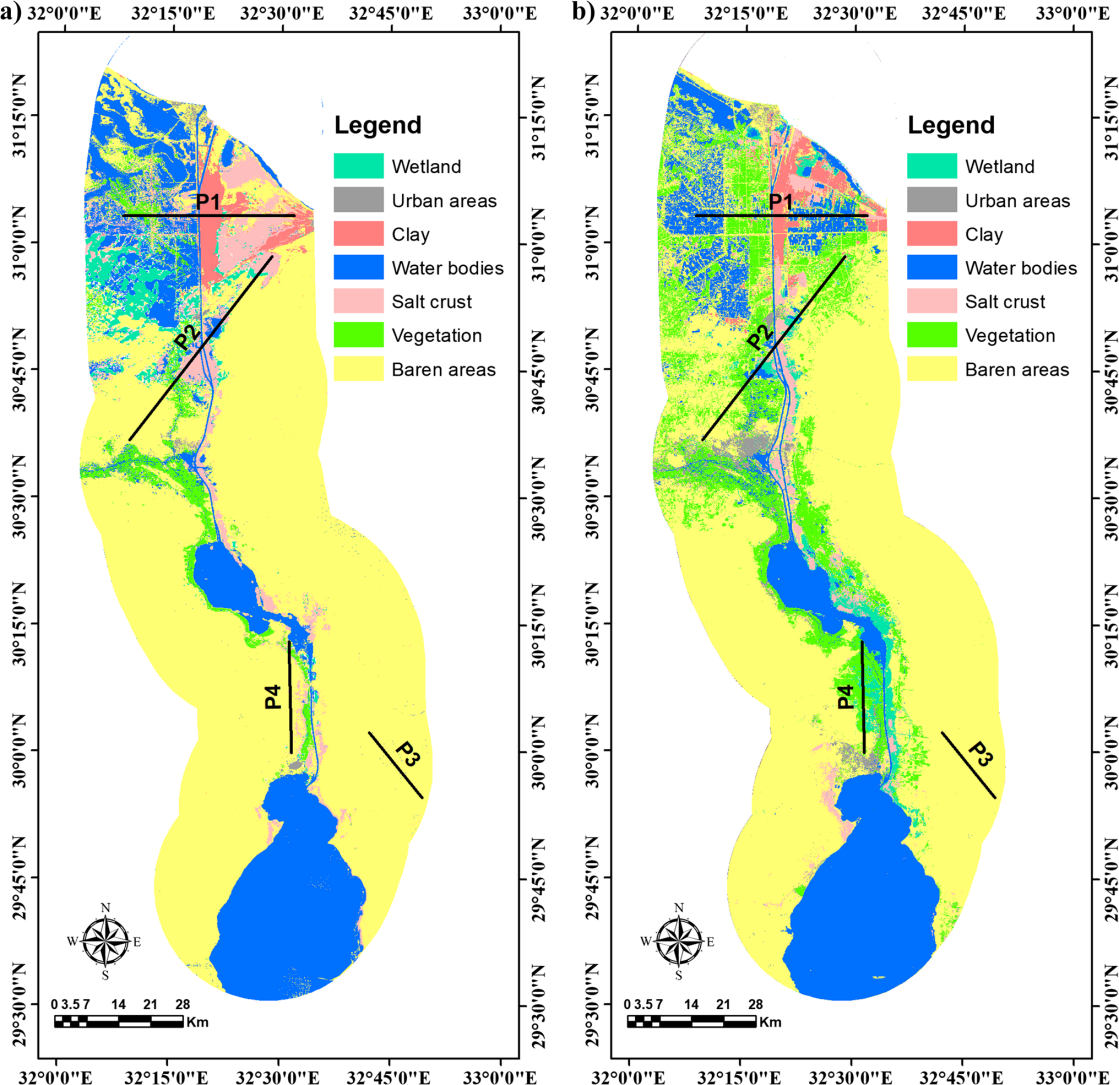
LULC classification of the study area for the years **a** 1984 and **b** 2018

The LULC classification has been categorized into seven classes, i.e., water bodies (waterlogged, lakes, sea, fish farm, and canal), urban (residential, industrial, and commercial zones), agriculture (high and low density) land, bare (sand dunes, sand sheet, and mountainous areas), wetland, clay, and salt crust (Fig. 5a and b). The results give an overall accuracy of the map and an accuracy for each class in the obtained classified map. Therefore, the pixels derived from the images were used to compare similar sites in this field. Furthermore, for validation, the classified maps’ random points (89 points) for TM 1984 and (92 points) for OLI 2018 have been collected from Google Earth images, geological and geomorphological maps. The overall accuracy percentage for classified images for the TM 1984 was 91.7, and for OLI 2018 was 90.1. The results’ reliability was derived with the help of the kappa coefficient, and the values of the kappa coefficient are 0.92 and 90 for the TM 1984 and OLI 2018. The pattern and spatial distribution of LULC changes were shown by preparing the multi-temporal LULC maps. The areal distribution of LULC changes was shown in Table 1.

**Table 1:** The results of change detection of land use/land cover between 1984 and 2018

Class	Surface area in 1984 (km^2^)	Surface area in 2018 (km^2^)	Difference (km^2^)	Average yearly rate of change (km^2^/year)
Wetland	275.44	261.01	− 14.43	− 0.42
Urban areas	26.21	151.1	+ 124.89	+ 3.67
Clay	204.04	199.34	− 4.7	− 0.138
Water bodies	2009.24	1935.67	− 73.57	− 3.06
Salt crust	567.75	278.26	− 289.49	− 8.51
Vegetation	306.67	1215.36	+ 908.69	+ 26.72
Baren areas	6530.87	5,877.06	− 642.97	− 18.91

**Fig. 6:**
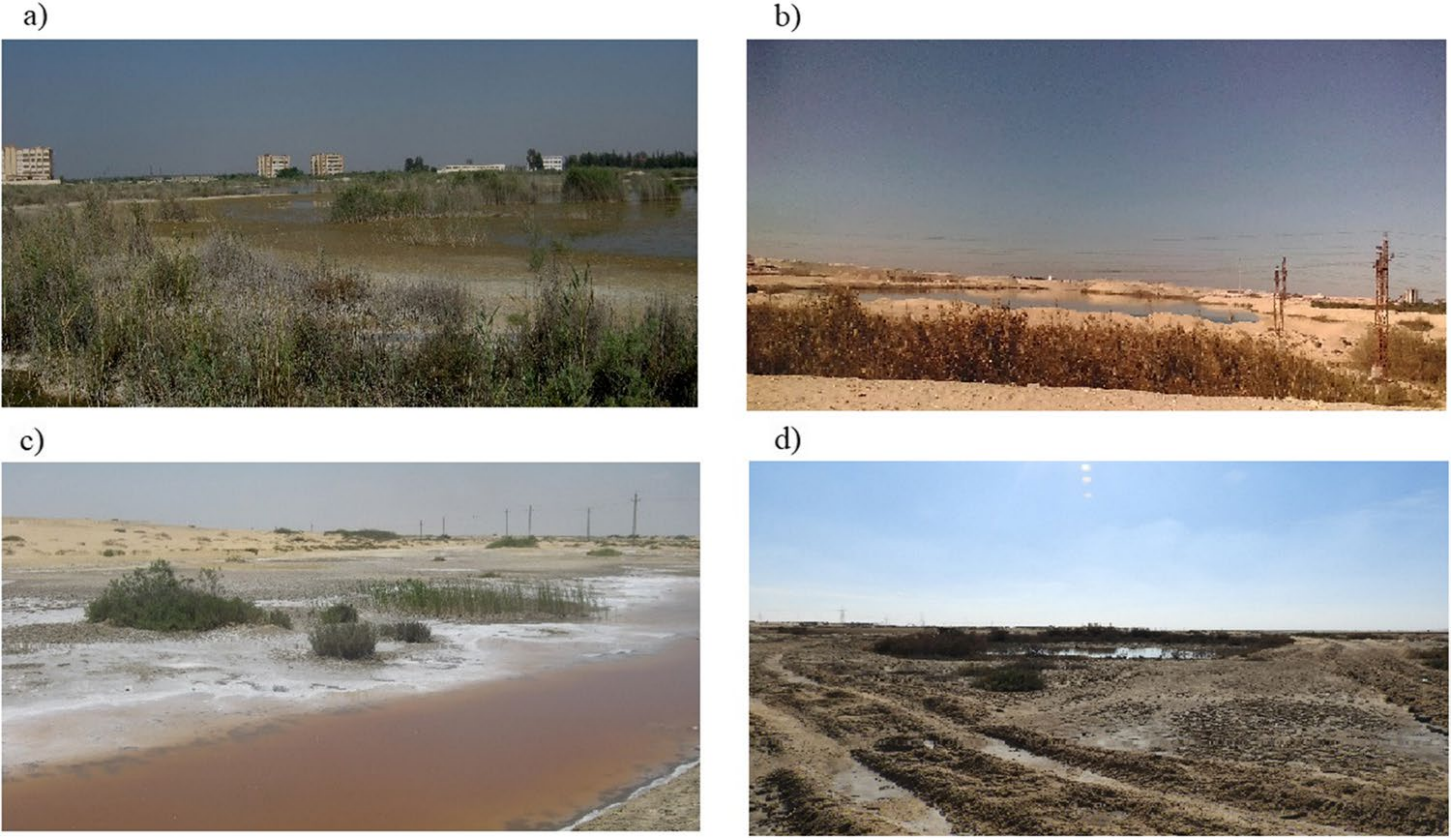
Some environmental and land degradation hazards found in SCR such as waterlogging and salinization at **a** El-Ballah area, **b** eastern bitter lake between sand dunes, **c** northern lowlands, and **d** along the Suez Canal

There is a significant increase in the urban area, including settlement and industrial areas, from 26.21 to 151.10 km^2^. Also, there was a significant increase in vegetation, including dense and scattered vegetation, from 306.67 to 1215.36 km^2^, while the salt crust decreased from 567.7 to 278.26 km^2^. Along with LULC changes, the rate of change has been calculated to depict which LU types are converting very rapidly to other LU types (Table 1). The obtained results indicated that the majority of the baren land was converted into agriculture and urban with an average rate of change + 26.72 and + 3.67 km^2^/year, respectively. Simultaneously, the clay and salt crust areas were reduced from 204.04 to 199.34 km^2^ and 567.75 to 278.26 km^2^ respectively, to transform into fish farms and vegetation land with an average rate of change − 0.138 and − 8.51 km^2^/year, respectively. Nevertheless, the wetland area declined from 275.44 to 261.01 km^2^ with a rate of change − 0.42 km^2^. The water bodies decreased in area from 1984 to 2018 by − 73.57 km^2^ and the change rate of − 3.06 km^2^/year due to the shrinkage of LM and Al- Malha Lake (Fig. 5a and b). Overall, the SCR’s development continuously led to some environmental issues. These issues were caused by uncontrolled rapid urbanization and land reclamation including residential, industrial, commercial, other concrete buildings, and agricultural activities. Therefore, most of these LULC developments lead indirectly to environmental and land degradation hazards such as increased salinization processes, pollution of water, and soil, and waterlogging (Fig. 6).

### Multi-temporal thematic thermal maps and their distribution

**Fig. 7:**
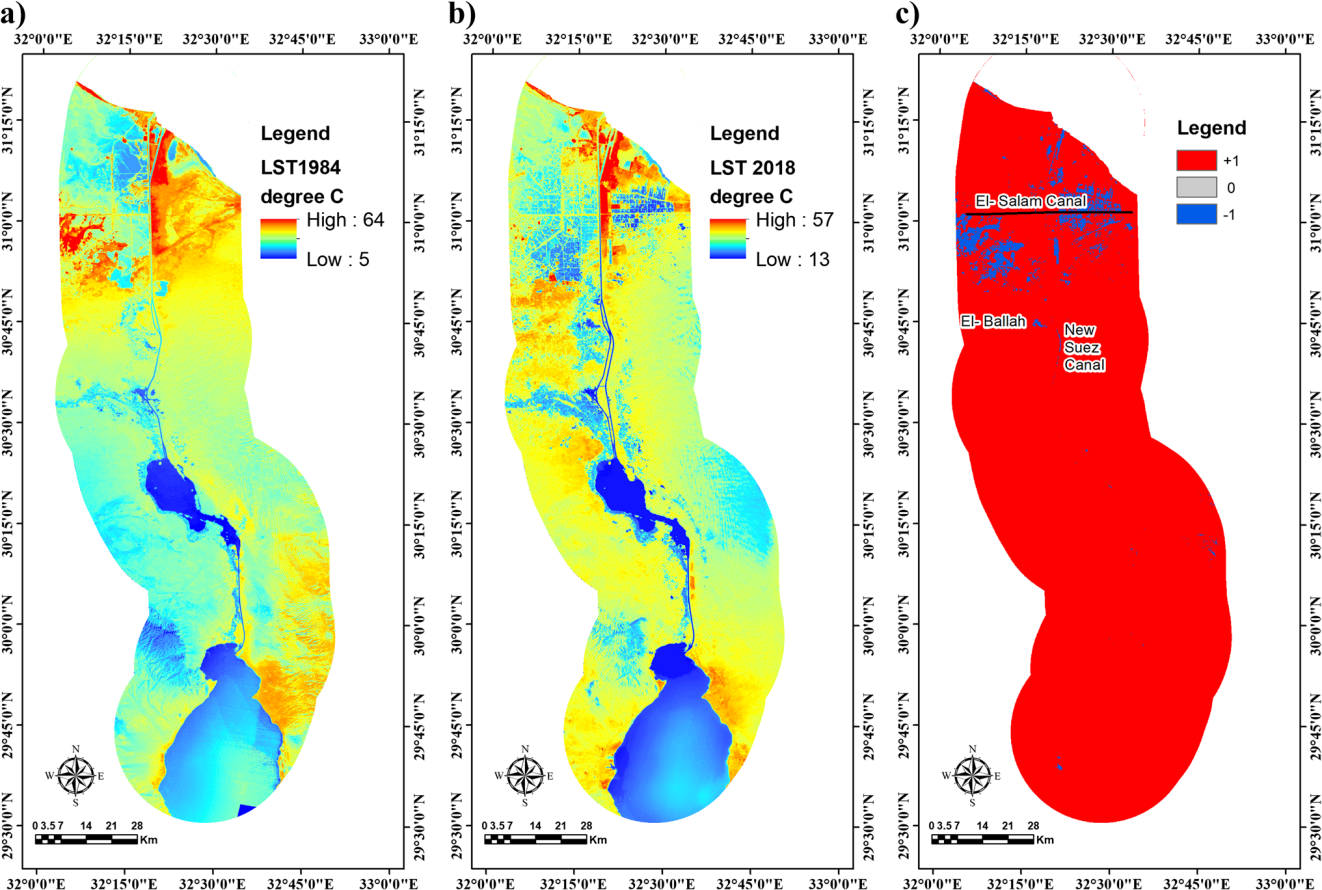
Calculated LST in degree centigrade of the study area for the years **a** 1984 and **b** 2018 and **c** the results of change detection

Figure 7 shows the calculated LST of the SCR. Temperature statistics related to each LULC class were obtained using zonal statistic tool in ArcGIS. Table 2 shows that the maximum recorded temperature of TM 1984 was 59.8 °C and a minimum temperature value of 5 °C (Fig. 7a). On the other hand, on OLI/TIRS 2018 (Fig. 7b), the highest temperature value recorded was 57.38 °C and the lowest temperature was 13 °C. Therefore, the mean LST of the SCR within 34 years has increased from 31.97 to 43.92 °C with an average rate of change of 0.352 °C per year (Table 2), which reveals that the area is facing many environmental changes according to LULC changes. Table 2 shows the estimated values of seven major LULC classes. It reveals that the clay, wetland, urban, and salt crust areas in 1984 exhibited the highest LST having a mean value of 45.7 °C, 41.43 °C, 35.46 °C, and 33.46 °C, respectively. However, baren land, water bodies, and vegetation have lower LST, with a mean value of 21.5 °C, 22 °C, 24.27 °C, respectively. Moreover, the statistically estimated LST values in 2018 show that the LST values of clay, salt crust, wetlands, and urban areas exceed other LULC classes with a mean value of 51.69 °C, 47.29 °C, 45.20 °C, and 44.90 °C, respectively.

**Table 2:** The results of land surface temperature changes in land use/land cover classes between 1984 and 2018

Class	Temperature ranges in 1984 (°C)	Temperature ranges in 2018 (°C)	Mean Temperature in 1984	Mean Temperature in 2018	Mean Rate of LST change
Wetland	12.48–57.88	31.26–56.05	41.43	45.2	0.11
Urban areas	8.6–53	31.24–56.51	35.46	44.9	0.27
Clay	18.59–59.8	34.52–56.88	45.7	51.69	0.17
Water bodies	5–48.73	13–57.38	22	35.37	0.39
Salt crust	7.5–55.9	31.38–56.76	33.46	47.29	0.4
Vegetation	9.75–42.89	13–57	24.27	42	0.52
Baren areas	5–48	33–49	21.5	41	0.57

On the other hand, water bodies show the lowest LST values having a mean of 35.37 °C. From 1984 to 2018, the agricultural and reclaimed land activities in SCR have been increased. The temperature value around dry saline soil, residential, industrial, roads, and concrete pavements become considerably higher than bare land. Results of LST change detection (Fig. 7c) showed that 97.089% of the study area about 9631.47 km^2^ has increased in temperatures (red color) except for the southern part of LM and Sahl El-Tina plain (blue color) in the west and east of the Suez Canal, respectively counting for 2.91% of the study area as a direct impact of El Salam Canal construction in 2003 and the addition of the new Suez Canal branch in 2014. El Salam Canal intercepted the groundwater flow to the northern area of LM and the sea, increasing the water levels in the southern area (Mansour 2015), consequently lowering its LST. On the other hand, the availability of water in Sahl El-Tina plain after 2003 has led to major humanmade activities (agriculture and fish farming) on this plain’s clay soil nature. The faulty agriculture water drainage has led to the rising of the waterlogging problem in this area. Consequently, the land use changes in the Sahl El-Tina plain have lowered the LST of this area. Also, the construction of the second Suez Canal branch in 2014 has lowered the LST in this area, confirming the power of land use changes over LST.

Finally, the agriculture expansion on El-Salheya plain of relatively higher land elevation from its surroundings has led to the infiltration and flow of irrigation water to the lowlands of the El-Ballah area, which acts as a discharge zone for the whole Ismailia governorate in the west of the Suez Canal (Mansour 2015), thus, Lowering the LST in this area (Fig. 7c).

### Relation between LST, LULC classes, and change detection of the land degradation hazards

Comparing the LST and the LULC classification images, a rapid change in LST was observed with the increasing LULC changes. Table 3 shows the results of LULC change detection and its effect on LST obtained by model builder tools in ArcGIS desktop 10.3. The wetland, clay, water bodies, salt crust, and baren areas classes had a negative change as they decreased in their total area in 2018. Urban areas and vegetation, on the other hand, had a positive change. Most of the wetlands in 1984 were converted to vegetation and water, resulting in a minor increase in mean LST for wetland-vegetation conversion (2. 81 °C) and a significant decrease in mean LST for wetland-water conversion from 43.32 to 37.75 °C. In four decades, 70.55 km^2^ of salt crust and 35.03 km^2^ of water were converted to wetland resulting in a large increase in mean LST from 33.17 to 45.24 °C and 23.80 to 44.64 °C, respectively. Only 12.82 km^2^ of wetlands remained unchanged. From 2018 maps, 104 km^2^ of baren areas, 12 km^2^ of water, and 11.8 km^2^ of vegetation were converted to urban areas. This conversion was responsible for the increase of mean LST to 45 °C. Also, the clay area was mostly lost to 26 km^2^ of salt, 23.2 km^2^ of vegetation, and 18.5 km^2^ of water. The mean LST change for the lost clay areas to salt was the highest and changed from 39.55 to 50.21 °C. As for the water bodies, 178.2 km^2^ was converted to vegetation, 35 km^2^ to wetlands, and 21.91 km^2^ to clay, particularly in the northern dried areas of LM. Their mean average temperatures increased from around 24 to 47 °C. Meanwhile, 124 km^2^ of salt, 58.26 km^2^ of wetlands, and 55.13 km^2^ of vegetation were converted to waterbodies. LST in these areas either decreased or slightly increased. The most effective LULC class changes on LST were the conversions from or to baren areas. Of baren areas, 630.5 km^2^ were converted to vegetation, rising LST from 30.82 to 43.57 °C. Vast baren areas were converted to equal areas of 146 km^2^ to salt and water. 104 km^2^ of urbanization was added to the baren areas rising the LST to 45 °C. Also, 184.33 km^2^ of water bodies dried out and changed to baren areas rising the LST to 43 °C.

**Table 3:** Zonal statistics and LST changes with respect to change detection of LULC classes between 1984 and 2018 obtained by model builder tool in ArcGIS

Class	Type of change	Other classes	Areas lost to other classes in 2018 (km^2^)	Areas gained from other classes in 2018 (km^2^)	Unchanged areas (Km^2^)	Mean LST changes for lost areas from 1984 to 2018 (°C)	Mean LST changes for gained areas from 1984 to 2018 (°C)
Wetland	-ve	Urban Clay Water Salt Vegetation	2.24 8.11 58.26 7.55 72.54	0.35 4.76 35.03 70.55 2.13	12.82	37.32 to 46.35 43.19 to 51.21 43.32 to 37.75 42.57 to 49.44 40.03 to 42.84	31.21 to 45.11 43.46 to 44.62 23.80 to 44.64 33.17 to 45.24 26.17 to 43.58
Urban areas	+ ve	Wetland Clay Water Salt Vegetation Baren areas	0.35 1.14 0.45 0.40 1.14 7.91	2.24 0.14 12.28 6.42 11.84 104.15	14.39		32.78 to 44.24 40.22 to 44.95 26.04 to 43.25 30.83 to 44.16 24.78 to 43.53 30.11 to 45.28
Clay	-ve	Wetlands Urban Water Salt Vegetation	4.76 0.14 18.54 26 23.27	8.11 1.14 21.91 32.37 0.53	85.59	35.95 to 44.64 32.67 to 47.09 35.73 to 37.53 39.55 to 50.21 35.10 to 43.39	43.19 to 51.21 46.59 to 51.59 23.71 to 49.23 32.95 to 50.85 27.05 to 47.60
Water bodies	-ve	Wetlands Urban Clay Salt Vegetation	35.03 12.28 21.91 20.74 178.20	58.26 0.45 18.52 124.04 55.13	1569.08	23.80 to 44.64 26.02 to 43.23 23.73 to 49.19 21.08 to 47.83 24.06 to 42.29	43.27 to 37.77 27.43 to 40.74 43.21 to 37.58 33.59 to 37.05 24.51 to 38.15
Salt crust	-ve	Wetlands Urban Clay Water Vegetation	70.55 6.42 32.37 124.04 105.15	7.55 0.40 26 20.74 1.10	75.05	33.18 to 45.24 30.82 to 44.16 32.99 to 50.81 33.59 to 37.04 34.43 to 42.73	42.47 to 49.40 43.49 to 49.01 47.30 to 50.22 20.98 to 47.70 24.02 to 48.76
Vegetation	+ ve	Wetlands Urban Clay Water Salt Baren areas	2.13 11.84 0.53 55.13 1.10 44.57	72.54 1.14 23.27 178.20 105.15 630.50	194.94		40.02 to 42.83 38.08 to 46.94 42.55 to 43.42 24.03 to 42.27 34.48 to 42.71 30.82 to 43.57
Baren areas	-ve	Wetlands Urban Clay Water Salt Vegetation	134.94 104.15 48.13 145.74 145.46 630.50	113.65 7.91 45.74 184.33 149.26 44.57	5303.815	32.07 to 45.47 30.11 to 45.28 41.84 to 50.67 29.99 to 39.09 33.82 to 46.83 30.82 to 43.57	42.08 to 45.29 41.18 to 49.25 43.85 to 46.13 26.60 to 43.09 34.28 to 44.99 26.67 to 43.49

**Fig. 8:**
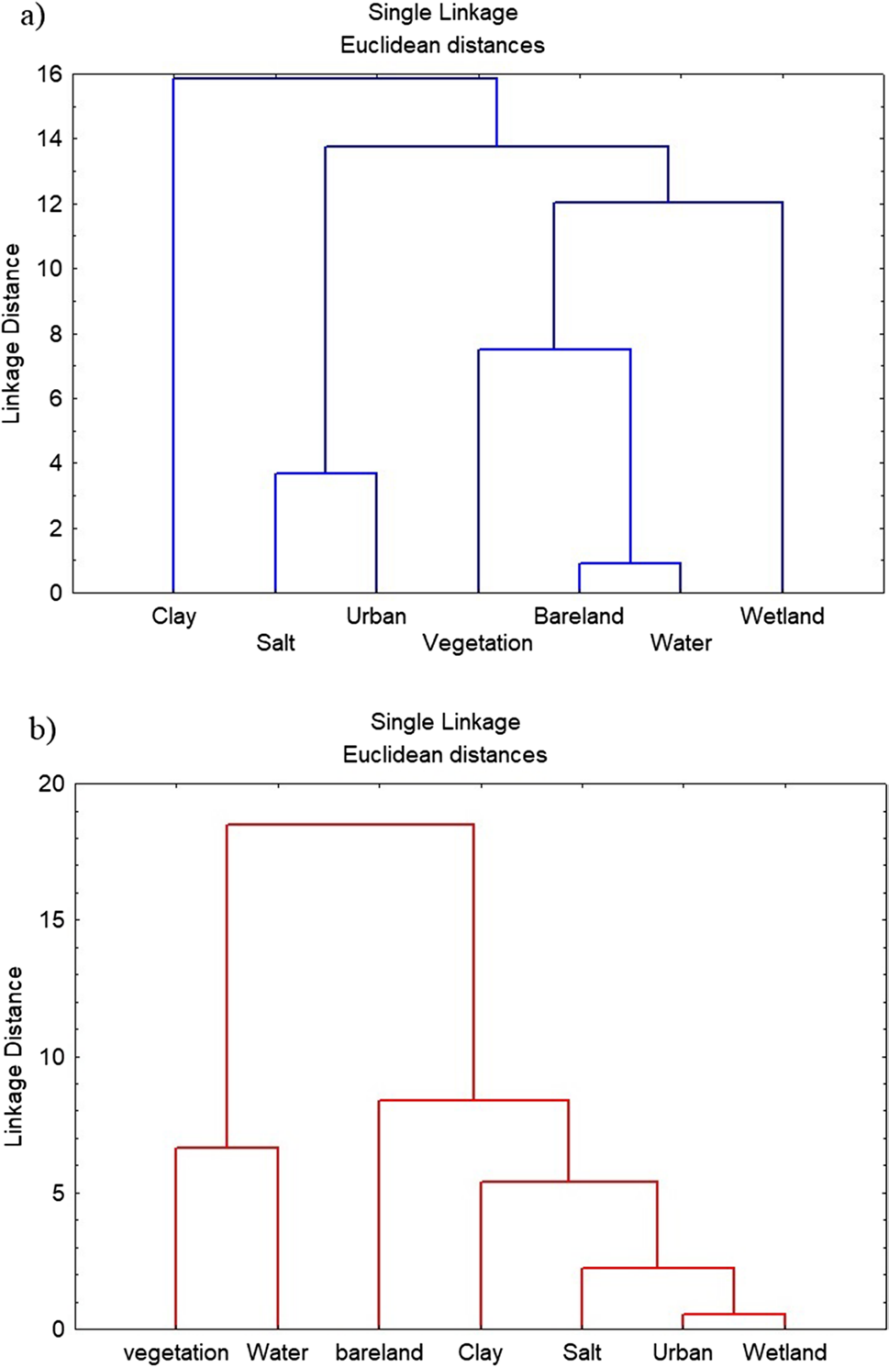
Cluster analysis of LULC classes in the study area for the years **a** 1984 and **b** 2018

**Fig. 9:**
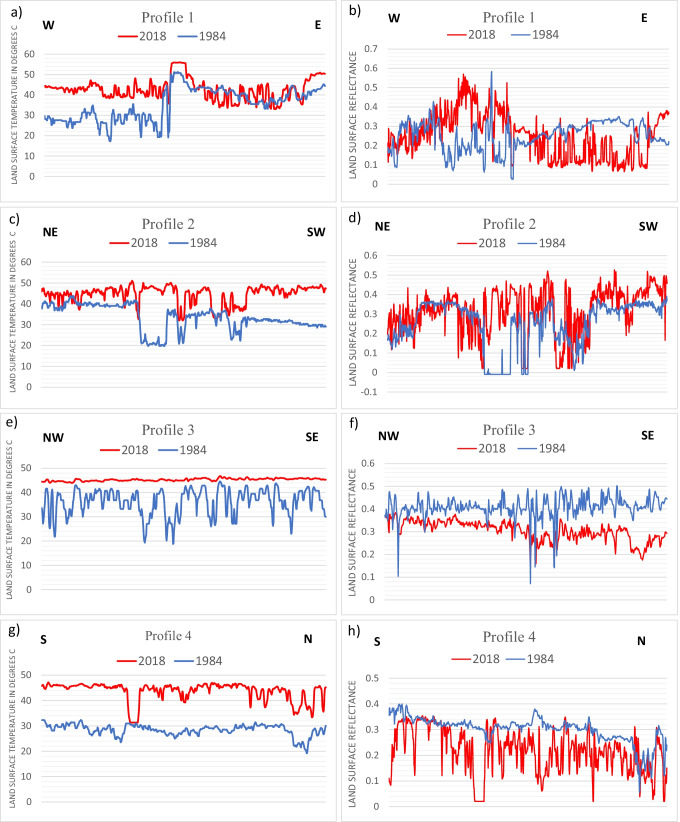
Cross-sections produced across the SCR area to represent LST (left) and LSR (right) variations between all major classes with the LULC transformation as a land surface reflection for 1984 and 2018. **a**, **b** Profile 1; **c**, **d** profile 2; **e**, **f** profile 3; **g**, **h** profile 4

Across the SCR area, cluster analysis between 1984 and 2018 LST ranges for LULC classes, and four cross-sections have been made to represent the variations of LST and land surface reflectance (LCR) between all major classes (Figs. 8 and 9, respectively). Cluster analysis was successfully used to classify LULC and determine the LST groups of distinct populations that may be significant in the land degradation for the obtained results of enhanced satellite data of 1984 and 2018. The *R*-mode dendrogram of the LST variables of the LULC in SCR was constructed and displayed in Fig. 8.

In 1984 (Fig. 8a), at linkage distance 10, four groups can be seen. The first cluster group was clay. The second cluster was salt crust, and urban areas and point to high LST values. The third group was vegetation with bare land, water bodies, and the fourth was wetland. The last two groups point to lower LST values. Figure 8a shows the natural response of different classes to LST values, as the salt crust and urban areas usually have the same spectral signatures and LST, hence clustered in the same group.

On the other hand, in 2018 (Fig. 8b), at the same linkage distance 10, two main clusters can be seen. The first cluster group is vegetation and water bodies, which reveals that the higher vegetation cover and the increase in the groundwater level help in lowering the LST. Furthermore, the second cluster group found between bare land and clay, salt crust, urban and wetlands may reflect the role of urbanization processes in SCR and the increase in the LST rate, particularly in the impervious surface and saturated saline soils and highlights the conversion happened between the different classes. Hence, Fig. 8b reflects the increase of human activities response on LST.

Four cross-sections were taken across the SCR in three highly reclaimed areas and one serine area (Figs. 5 and 9). Generally, the cross profiles reveal changes in the LST and LSR values.

In profile one from E-W, in the northern lands of LM and Sahl El-Tina plain (Fig. 9a and b), It was found that the salt crust, clay, and wetlands on the eastern side of the Suez Canal were transformed into fish farms and agricultural areas. On the western side of the Suez Canal, the water body of LM, bare land, and wetland were transformed into agricultural, industrial, and fish farms. According to these land transformations, it was observed that the LST of the eastern side were within the same temperature ranges but showed some displacement in LST values due to the interference between the different LULC classes. On the other hand, the LST temperatures of the western side showed an increase in LST values due to the drying of northern LM, i.e., a decrease in its water content and an increase in its salinity. The LSR values in this profile changed drastically due to the significant LULC changes.

Profile two from NE-SW (Fig. 9c and d) showed the effect of groundwater discharge zones reallocation due to vegetation expansion on the LST values. In the NE of the Suez Canal in this area, there was a large discharge zone collecting groundwater in 1984, which was completely dried out in 2018 with huge temperature variations. On the western side of the Suez Canal, a permanent discharge zone was observed in 2018 in El-Ballah area owing to the extensive agriculture expansion in the higher lands of El-Salheya plain with well-drained soil.

Profile three from NW–SE shows the increasing LST values of serine baren land from 1984 to 2018 (Fig. 9e and f). These changes in LST value may be due to the increase in the seismic activity and sand dune migration that covered the dried natural drainage distributaries in this area that showed some water content in 1984. The LSR of this profile confirms this water content in 1984 and the change of surface material (sand dune migration) in 2018.

Finally, profile four from N-S indicates the increasing and altering LST values of vast baren areas in 1984 at south bitter lakes (Fig. 9g and h). It was converted in 2018 into dense vegetation, waterlogged, wetland, and urban classes. The baren areas recorded a maximum of 35 °C in 1984. The maximum value of the baren land LST was raised to around 45 °C. Overall, it can be observed from (Fig. 9) that the LSR in all profiles except profile three shows some interference between the different classes in 2018, which coincides with the cluster analysis results in Fig. 7 reflecting the LULC changes (merge between classes) and their effect on LST.

### Detecting and monitoring the hydro-environmental impact and land degradation

**Fig. 10:**
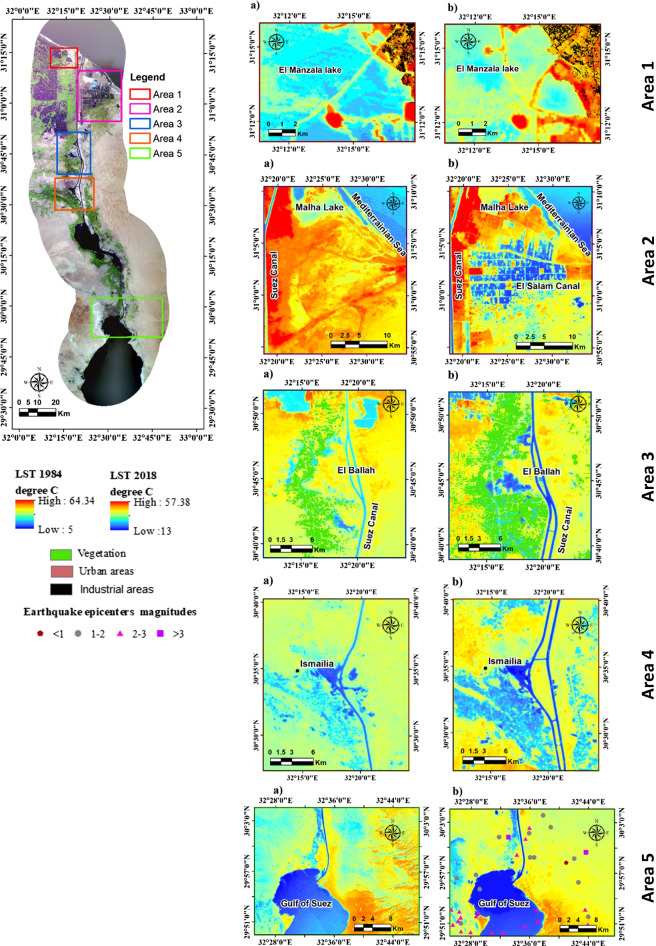
LST of five hot spots taken across the SCR for **a** 1984 and **b** 2018

Visual interpretation of the resulted maps depends on RS image characteristics and prior knowledge of the investigated area. Fieldwork and other ancillary geological and environmental data were used to help identify and map land degradation. The following section will compare five different hot spots (Fig. 10) from the enhanced LST and LULC multi-temporal satellite between 1984 and 2018 to identify the environmental and land degradation in SCR. These degradations include natural, human activities, particularly agricultural, industrial, and urban activities, water pollution, groundwater level rising, salinity increase, sand dune migration, and seismic activity.

The most observed land degradation impacts on the environment in the SCR resulted from the construction of El Salam Canal in 2003 represented by hotspots one and two (Fig. 10). It resulted in changing the natural environment of LM and Sahl El-Tina plain. The LM southern part was flooded with water resulting from the cut of natural groundwater flow to the north by El Salam Canal, changing the area to cultivated lands, waterlogging and fish farms (Mansour 2015). This resulted in decreasing the LST in these areas from 43 to 37 °C. On the contrary, the LM northern part dried out, making it a perfect location for industrial, agricultural, and domestic wastes. Hence, the water quality deterioration of the northern part of LM changed the LST values dramatically between 1984 and 2018. The LST values increased from 28 to 45 °C near the industrial and urban zones. The vast amounts of contaminated wastewater and accumulated bottom sediments near these sites contain heavy metals that changed the pattern of surface water temperature to circular rims around these areas. The contaminated water and sediments’ heat pockets are indicated by the shades of blue and yellow colors in the LST (Fig. 10). These results agree with Arnous and Hassan (2015); their findings revealed that the water and sediments of LM constitute an ultimate sink for heavy metals in the LM aquatic system.

At Sahl El-Tina plain hot spot two (Fig. 10), most of the canal’s water was used for flood irrigation practices introducing the concept of fish farming and waterlogging in the area and lowering its LST values. The detection and the monitoring of the results of environmental impacts and land degradation were based on the change detection of LST values and agreed with the results of Arnous et al. (2017), El-Rayes et al. (2017), and Moubarak et al. (2021). The change in the LST values may be attributed to the dominance of saline soils and baren lands and to the natural evaporation process acting on the saturated soils, leading to increased soil salinity and increasing LST values. Salt crust is mostly located in the middle and the coastal zones under the impact of evaporation of the seawater and waterlogging along the ground surface. Moreover, the sand dunes class's LST values in 1984 were 33–37 °C, which increased after 34 years to 46 °C due to migrations of sand dunes covering the vast area of saline soil and clay classes. The area at Malha Lake in 1984 was mostly covered by saline water bodies and saturated soil with LST values of 5–32 °C, which increased to 46–54 °C after being transformed into baren and saline soil after backfilling and draining the primary area of the Malha Lake.

The land degradation effect resulting from vegetation expansion was observed in hotspots two, three, and four (Fig. 10). This increased the LST from around 24 to 43 °C and is mainly due to evapotranspiration, flood irrigation practices, poor drainage, shallow clay lenses, altered groundwater recharge and discharge zones, and increased soil salinity. It was evident that LULC changes, especially those related to the surface soil moisture, varied extensively at hot spot 3 and was confirmed by several studies such as Mansour (2015), Arnous and Green (2015), Arnous et al. (2015), and Hassan et al. (2019). The least LST values were identified in waterlogged areas 5–24 °C in 1984 and 13–32 °C in 2018, especially in El-Balah area the due to changes in the source and water quality with the groundwater level rising, increasing the LST of wet saline soil to 29–32 °C and LST of the fish farms (13–32 °C in 2018). In contrast, the cultivated areas are characterized by low to moderate LST values due to the change in crop type and irrigation systems. The extensive land reclamation and agricultural activities caused the appearance of many hydro-environmental problems such as waterlogging and soil salinization due to irrigation and rising waterlogged sites by seepage towards low topographic areas. The highest LST values were detected in baren soil (50–57 °C in 2018), particularly at El-Salheya plain, due to low vegetation density and well-drained soil. Some sites located on the eastern side of the new Suez Canal suffered from a change in the LST values from 33–37 °C in 1984 to 46–49 °C in 2018 due to the increase in evaporation of the heavily moist surface soil. It is worth mentioning that in 1984 there was a large lake east of the Suez Canal navigation route with temperature ranges between 21 to 24 °C. This lake dried out due to the increase in vegetation crops which are irrigated by mixed fresh and groundwater (pumping from wells dug in the area). Also, the loss of vegetation to urbanization rose the LST even higher to 49 °C.

Speedy urbanization driven at or around both Ismailia and New Ismailia Cities, hotspot four (Fig. 10) by population growth and economic development had severe and extensive modifications to the land surface, causing the replacement of natural surfaces such as vegetation with impervious surface materials such as concrete, asphalt, and buildings. It also transformed the baren land and agricultural area to dry and saturated saline soil and waterlogged areas (Fig. 10). The widespread LULC transformations have created ecological and environmental problems at multiple scales in this hot spot. The LULC of this area in 1984 shows that the urban area of Ismailia City has LST values ranging from 25 to 32 °C (Fig. 10). After 34 years, the rapid urbanization such as residential, industrial, parks, and institutional LU and land reclamation led to increasing the LST values from 40 to 57 °C in 2018. In addition, the baren land (LST values 29–32 °C in 1984; 46–57 °C in 2018) and agricultural areas (LST values 21–24 °C in 1984; 13–35 °C in 2018) were transformed into extensive agricultural actions with decreasing LST values. Furthermore, the saline areas were transformed into waterlogged areas in the eolian plain and the windblown deposits, particularly at the southern of Wadi El Tumilat due to the continuous groundwater level rising. The same behavior was observed around the new Suez Canal due to the increase in surface soil moisture. The above results agree with many literature (Ghodeif et al., 2013; Arnous and El-Rayes 2013; Arnous et al. 2015; Mansour 2015; Arnous and Green 2015; Hassan et al. 2019).

Hotspot five (Fig. 10) shows the land degradation impacts of urban, agriculture, sand dune migration, and seismic activities. The change of the LST values may be attributed to the dominance of saline soils and baren lands and to the natural evaporation process acting on the saturated soils, leading to increased soil salinity consequently increasing the LST values from 42 to 53 °C. Salt crust is mostly located along the coastal zones of the Suez Canal’s eastern side, leading to the spreading of the seawater and waterlogging along the ground surface associated with evaporation and impacting the LST change in values from 33–42 °C to 46–53 °C. The conversion of baren land to agricultural lands and new urban extension, particularly at Suez City, led to the appearance of numerous waterlogged locations and increased the saturated saline soils nearest and close to the cultivated lands. The integration between LULC, LST maps, and overlaid seismic data revealed that the study area was subjected to tectonic activity and active faults and led to the detection of some neo-tectonic. The accumulation of earthquake epicenters from 1997 to 2017 also raised the LST and its effect is more shown, especially in baren areas where LST increased from 25 to 46 °C.

**Fig. 11:**
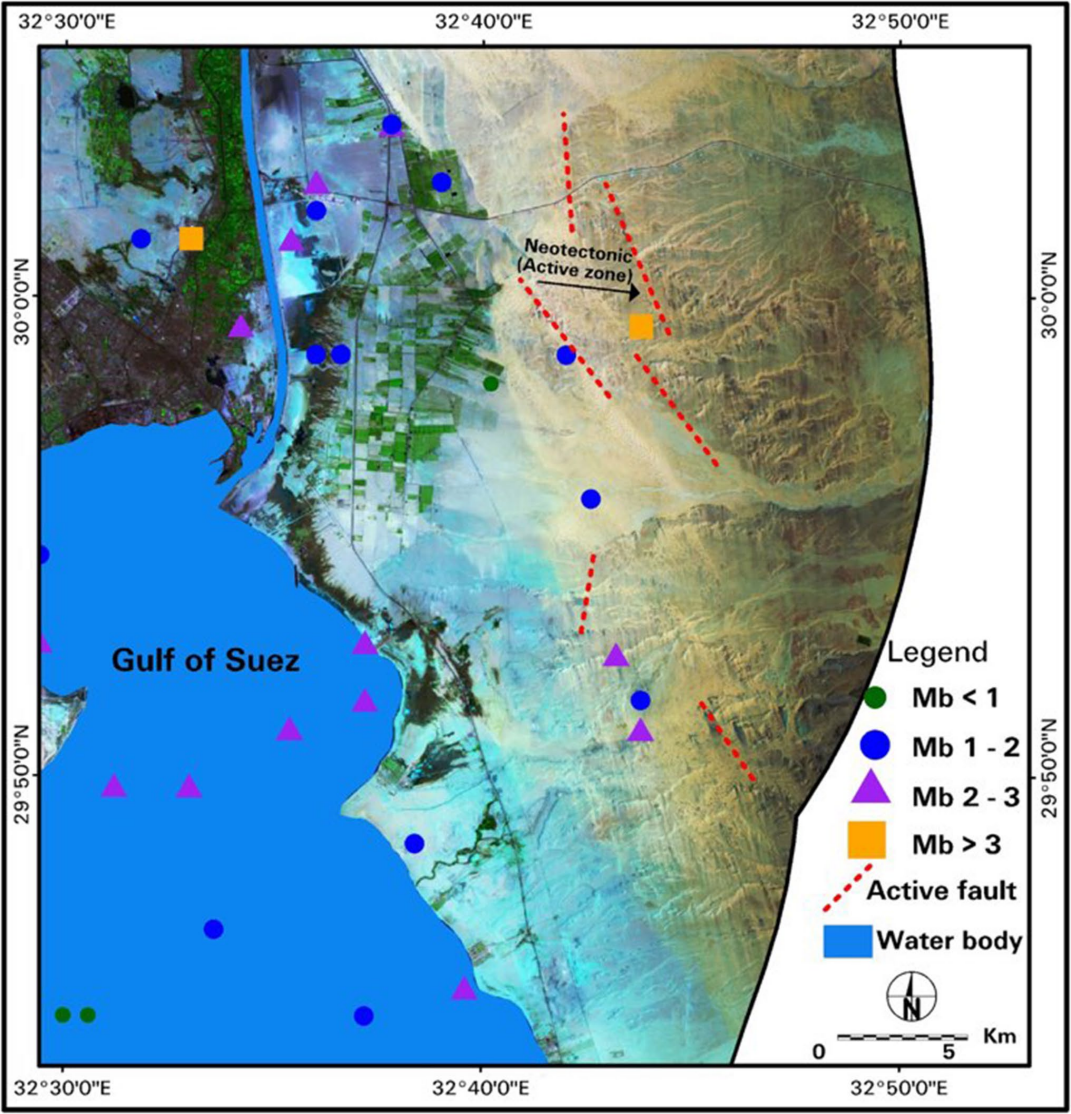
Integrated remote sensing data and seismic data to show some prints related to the seismic activity and neo-tectonic

These neotectonics features could be detected and monitored through the analyses of the LST multi-temporal data. The fault plain may offer a perfect avenue to moisture or vegetation growth and may form specific drainage patterns easily detected on the enhanced satellite data. According to the enhanced satellite data results and the epicenters data in this hot spot, there is a sudden change of the course of the drainage system with displacement inferring to structural control of the area and the overlay with epicenters around the area. These integrated and overlaid data revealed that the study area was subjected to tectonic activity and active faults based on the detection of some neo-tectonic features and the tracing of some structural tectonic active faults in the SCR area (Fig. 11). The western side of the Gulf of Suez also showed increased LST from 25 to 46 °C with accumulated epicenters especially in baren areas.

**Fig. 12:**
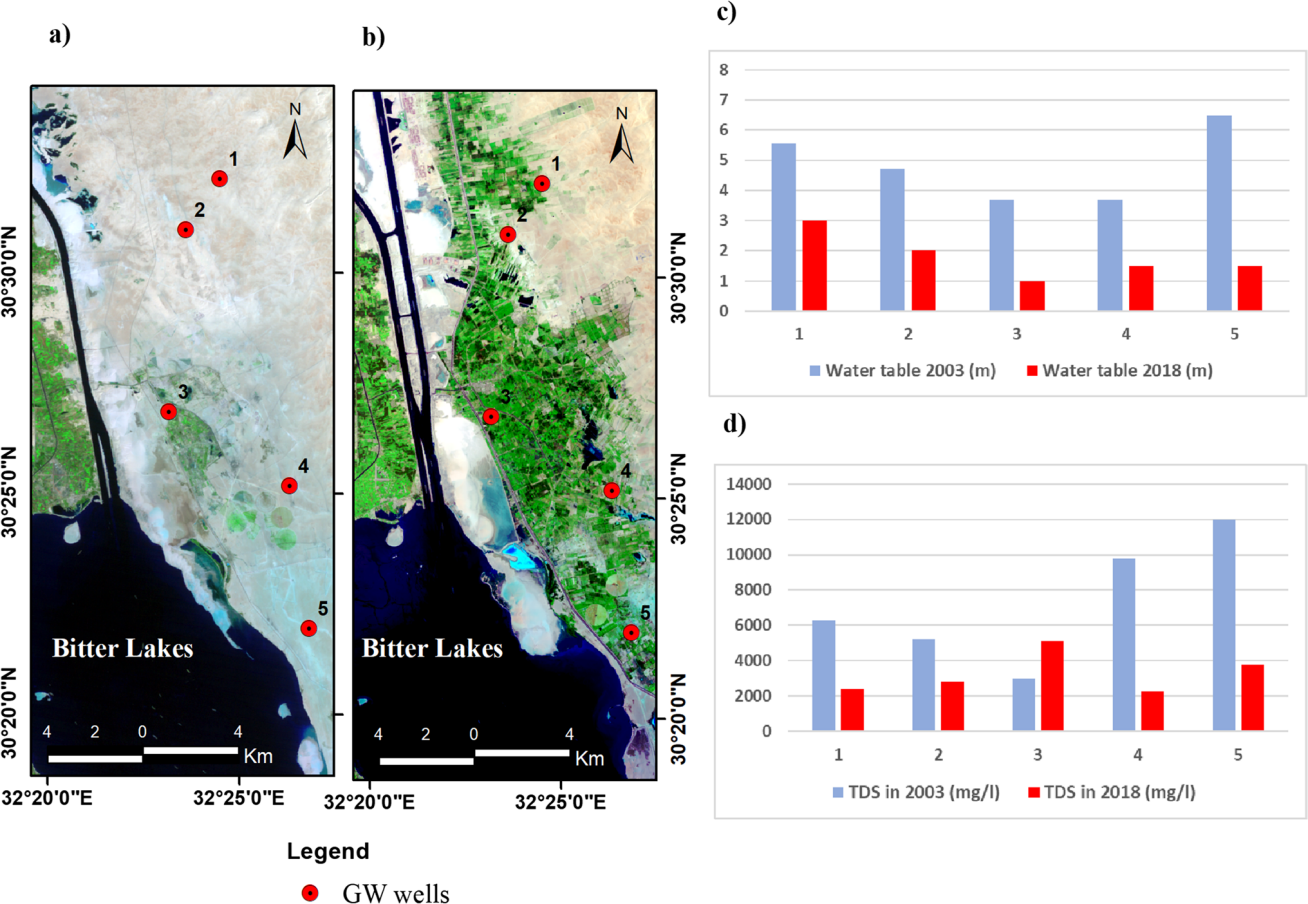
The change in groundwater levels (**c**) and salinity (**d**) across the SCR area from 2003 (**a**) to 2018 (**b**)

Table 4 and Fig. 12 sum up the relation between increased agriculture and waterlogging (groundwater rising). Increasing the vegetation cover in the area East of the Bitter Lakes decreased the groundwater levels from the year 2003 to 2018 due to the mixed irrigation between surface (Sinai Canal) and groundwater and over pumping from wells. However, some areas suffered from waterlogging due to the shallow clay layers underneath (return flow) which prevents the vertical percolation of water and increases the surface water salinity due to evaporation. Deeper groundwater has lower salinity and is less affected by the evaporation process, where the maximum effective evaporation depth in this area is about 0.6 m below the ground surface (Geriesh 2004).

**Table 4:** The change in soil water level (perched condition) and salinity between 2003* and 2018

Sample	TDS in 2003 (mg/l)	TDS in 2018 (mg/l)	Water table 2003 (m)	Water table 2018 (m)
1	6300	2400	5.55	3
2	5200	2800	4.7	2
3	3000	5120	3.7	1
4	9800	2260	3.7	1.5
5	12,000	3800	6.5	1.5

^*^Geriesh, 2004

## Conclusion

The present study identifies and assesses a fundamental hypothesis extensively used in geospatial technology attributing LST anomalies to LULC where, the LULC changes are the driving forces of the land degradation, producing major thermal changes. The multi-temporal Landsat TM + 5 and TIRS + 8 satellite data dated 1984 and 2018, statistical analysis, and fieldwork succeeded in detecting and mapping the multi-temporal LST, LULC changes, and their land degradation impacts on the SCR. The SCR was classified into seven major LULC classes namely, water bodies, urban areas, vegetation, baren areas, wetland, clay, and salt. The results showed that, in the last 34 years, the SCR suffered from a change in land under build-up, vegetation, wetland, salinization, sand dunes, and water bodies classes. These have shown significant expressions due to human and natural activities. LULC and LST change detection and mapping results revealed that only vegetation and urban areas had a positive change, i.e., increased in their areas. Moreover, 97% of the SCR witnessed LST rise during this period with an average rise rate of 0.352 °C per year. The most effective LULC class changes on LST were the conversions from or to baren areas. Baren land has shown a considerably high conversion rate and decreased territorial extent where, 630.5 km^2^ and 104 km^2^ of baren areas were converted to vegetation and urban areas rising the LST to 43.57 °C and 45 °C, respectively. Also, 184.33 km^2^ of water bodies dried out rising the LST to 43 °C. The spectral signature, LST profiles, and the statistical analyses studied the association between LST- and LULC-deriving factors. LST, fieldwork, statistical analyses, and their interpretations indicate that the clay class with the salt crust and urbanization shows the highest LST values. The cluster analysis of 1984 LST map showed the natural response of different classes to LST values. While the cluster analysis of 2018 LST map reflects the increase of human activities response on the LULC classes’ LST. Some interference between the different classes was observed as all high LST classes were clustered in the same group. The same interference was observed within the spectral profiles taken in 2018. The SCR changed areas impacted the LST and are associated with land degradation and hydro-environmental impacts such as groundwater level rising, salt accumulation, active seismic fault zones, sand dune migration, water pollution, and urban and agricultural activities. Usually, these impacts contribute to the change in LST patterns and levels. Environmental land degradation impacts should be taken into consideration before the land use planning of any area.
